# Supplementary material for: The effects of sex and outcome expectancies on perceptions of sexual harassment
Source: PLoS One. 2021 Dec 15;16(12):e0261409. doi: 10.1371/journal.pone.0261409 (PMC8673621; doi:10.1371/journal.pone.0261409)
Supplement: S1 Table — (DOCX) [file pone.0261409.s001.docx]

**S1 Table**

*Correlations (r) Between Individual Differences and Harassment Perception and Positive and Negative Outcome Expectancies by Sex*

|  | **H** | | **POE** | | **NOE** | |
| --- | --- | --- | --- | --- | --- | --- |
| **Trait** | **Men** | **Women** | **Men** | **Women** | **Men** | **Women** |
| Machiavellianism | -.18 | -.03 | .22 | .14 | -.25 | -.12 |
| Narcissism | -.11 | -.03 | .30 | .17 | -.25 | -.09 |
| Psychopathy | -.11 | -.08 | .29 | .15 | **-.35** | -.09 |
| Hostile sexism | -.22 | .13 | **.45** | .25 | **-.37** | -.19 |
| Benevolent sexism | -12 | .08 | **.38** | .25 | **-.32** | -.26 |
| Agreeableness | .06 | -.13 | -.00 | .29 | -.04 | -.15 |
| Conscientiousness | .09 | --.11 | -.17 | -.01 | .20 | .03 |
| Extraversion | .06 | -.15 | -.19 | .16 | .09 | -.17 |
| Neuroticism | -.16 | .08 | -.02 | .14 | .05 | -.14 |
| Openness | .12 | -.14 | -.18 | .06 | .10 | -.15 |
| Intrasexual Competitiveness | -.10 | .08 | .18 | .26 | -.27 | -.11 |
| Sadistic tendencies | -.13 | -.06 | .18 | .24 | -.24 | -.06 |
| Rape Myth Acceptance | -.18 | -.06 | **.43** | **.39** | **-.36** | -.20 |
| Sexual harassment experience (*n* = 52 men, 70 women) | .24 | .10 | .00 | -.17 | -.03 | .04 |
| Sexual harassment engagement (*n* = 15 men, 14 women) | -.16 | .17 | .22 | .06 | -.17 | -.03 |
| Age | .29 | **.32** | .02 | **-.35** | .01 | .15 |

Correlations in bold are significant to the p < .05 level following Bonferroni correction

H = harassment perception, POE = positive outcome expectancy, NOE = negative outcome expectancy
