# Supplementary material for: The effects of sex and outcome expectancies on perceptions of sexual harassment
Source: PLoS One. 2021 Dec 15;16(12):e0261409. doi: 10.1371/journal.pone.0261409 (PMC8673621; doi:10.1371/journal.pone.0261409)
Supplement: Attachment — Submitted filename: Response to reviewers.docx [file pone.0261409.s003.docx]

**17/09/2021**

**Response to reviewers**

***Thank you for giving us the opportunity to revise our manuscript, titled “The Effects of Sex and Outcome Expectancies on Perceptions of Sexual Harassment”. We have tried our best to address all concerns raised by the reviewers and believe the manuscript has been substantially improved as a result.***

COMMENTS TO THE AUTHOR:

**Associate Editor:**

A highly-qualified peer reviewer submitted excellent comments on the paper and suggestions for improvement. As academic editor, I reviewed the paper independently. It is clear that this paper is well written and represents a potential contribution to the literature on sexual harassment and positive/negative expectancies, particularly in online dating contexts. At the same time, the reviewer considered the study underdeveloped in terms of the conceptual framework and the literature review on which it is based. The concerns by the reviewer and myself include lack of validation work on the vignette paradigm used, conceptual framework that does not integrate some of the most relevant literature out there, and lack of clarity as to the unique contributions and advancement of knowledge. I do think the focus on positive and negative expectancies borrowed from other literatures (e.g., substance use) and the online dating context could be leveraged in a way that this paper could make an important contribution. For this reason, I would like to extend an opportunity to substantially revise the paper for resubmission, if the various critiques in this letter and in the review can be adequately addressed in a revision. This may be a high bar, as such a revision would involve a reconceptualization of the paper and major re-analysis.

Other comments to address in revision:

The exploratory analyses are under-developed and justified, and seem to be beyond the scope of the conceptual framework

***As suggested, we have moved away from focusing on an evolutionary psychological concept to a broader framework that accepts sexual harassment is likely multidimensional. We have added more literature to support our examination of positive and negative outcome expectancies (pp5-6). We have also expanded the exploratory analyses to examine individual vignettes and enhanced the discussion around associated traits (p23 for discussion of these). We believe this has greatly improved the overall quality and clarity of the manuscript.***

Too much time spent trying to justify vignette methodology you used and dismissing other methodologies, instead of describing validation of the vignettes

***We have now included our reliability analysis of the vignette scores (p10) and explained in the introduction why we have chosen a novel vignette measure for our study whilst acknowledging the merit of existing measures (p7).***

Information is needed on the types of websites and other locations in which the study was advertised: at a university, locally, or anywhere on internet?

***This oversight has been corrected and we now state where our study was advertised in the methods section (p9).***

The various analyses should use a multivariate framework, that include interactions with sex/gender, such as in the hierarchical regression and exploratory analyses

***Thank you for the suggestion. This is something that we had considered when drafting the paper. However, we felt that examining the sexes separately from the outset was more in line with the nature of the study and the goal of investigating sex differences. There is also precedent for this approach in the sex differences literature as separate analyses of men and women can reveal qualitatively different relationship patterns between variables that simple examination of mean differences often overlooks (Thomas et al., 2021) We now include a better explanation of this throughout the introduction (pp 3-8) and hope that you will deem our reasoning to be satisfactory.***

The PxN interaction is decomposed in a way that makes interpretation difficult to translate into real world behaviors

***We have added a paragraph in the results explaining the data presented in the simple slopes plot including a worked example which explains what the moderation effect means for responses at the DV level (p20). We agree that these findings, because they are not behavioural, are hard to translate into real world terms However, we hope that our further explanation helps readers get a better feel for the impact of positive outcome expectancies as a moderating force.***

A power analysis is needed to justify the sample size adequacy, especially to detect interactions by sex and PxN interactions (or three way interactions)

***Thank you. We have now added this to the participants section (p9).We have also added Cohen’s d values to our results section when discussing sex differences found in individual vignettes and aggregate harassment perception scores***

About 1/3 of your sample identified as non-heterosexual and all your vignettes involved male on female harassment – how does this affect your results?.

***A very good point, one that we checked and have noted in the results section as no qualitative differences were found (p19).***

**Reviewer #1:**

The authors use evolutionary psychology as a framework to understand sexual harassment and defining sexual harassment and perceptions of consequences between men and women. Using a quasi-experimental vignette design, they find some overlap between men and women, but also differences with regard to the likelihood of defining behavior as harassment and some of the correlates related to expectations.

There is certainly a voluminous literature that draws on evolutionary psychology to explain sexual violence, but the authors’ rationale and the methodology in this study do not seem to warrant that narrow framework given they are not actually testing motivation from an evolutionary psychology framework. In tailoring their study to an evolutionary psychology framework, they omit much of the relevant research in this area which potentially tempers the contribution of this study.

***Thank you for this recommendation. We have revised the introduction to discuss various theories of sexual harassment behaviour, removing the focus on evolutionary psychology. We believe discussing these theories better demonstrates how our research further contributes to this subject (pp 3-8).***

More conceptual development is needed by the authors. Elaboration on the omitted literature and its relevance to the authors study is provided below.

Although the authors use analytic methods that do not assert temporal order, the authors repeatedly state that by including expected outcomes and the perception of a behavior as harassment, that they are providing evidence that individuals define harassment based on the expected positive and negative outcomes of the behavior. However, using this survey design, the authors cannot ascertain temporal order. Why would expectations of outcomes (meeting up or reporting) occur prior to the interpretation of the situation as harassment? It is equally (and perhaps more likely) that individuals interpret the behavior as harassment and that defines their expectations for how the actors will behave. Indeed, drawing on social psychological literature, we would expect that the cognitive order would proceed this way. Ruback and colleagues (1984) theoretical and empirical work on reporting victimization to police outlines a three-stage model where interpretation of the event as problematic is the first step to reporting. This is not unlike the authors dependent variable of reporting harassment. Likewise, the work of Latane and Darley (1969) and the decades of research that has come from that study has asserted that interpreting a situation as problematic is the first step in third-party intervention. Situating their analysis in that framework would necessitate engaging with a body of literature that is not included in this study, but one that is necessary to justify the contribution given their analysis is similar analytically, but not conceptually, to prior work in this area.

***This is a very good point. We have now included a discussion of temporal order (whilst acknowledging that our study was not able to ascertain this information) and referred to the most likely presentation as outlined in the literature mentioned above (p26). We have also elaborated on our reasoning for the possibility of an alternative temporal order (outcome expectancies influencing harassment judgements). (p26).***

The authors attribute the idea that anti-harassment programs do not work because they are not tailored to men. This is not exactly what a lot of the prevention science research on sexual violence finds. Even if tailored, and sometimes especially if tailored, these programs can create a backlash effect. It is not because programs are failing to identify the underpinnings of sexual assault as the authors argue, it is that men do not want to be labeled as perpetrators and when they are subjected to training, they engage in victim blaming as a means of cognitive self-preservation. Dobbin and Kalev have done some excellent work in this area. Likewise, Tinker has also examined backlash as a function of anti-harassment policies. It is unclear how this study specifically relates to sex-specific programming and addresses or refutes the problems found in prior literature.

***Thanks to this comment we realise we have not made our intended points clear. We have rephrased and reorganised this section of the introduction with the hope that we have now better explained (pp 3-4). We intended to say that, while interventions are often sex-specific, they are not tailored to men based on their unique sexual psychology, but, as the reviewer says, are tailored to men in the sense that they assume that they are a potential perpetrator. We agree wholeheartedly with the view that this is a counterproductive approach and would like to promote an intervention that first understands those most likely to harass and takes a prosocial goal-orientated approach. It is these goals that we believe may be sex-specific. We have discussed this in relation to some of the literature provided by the reviewer (p 4).***

Likewise, the authors suggest that their study has important implications for programming. Namely, it will provide evidence that sex-specific programs are necessary. One of the conclusions from their analysis is that sex-specific programming may be helpful. Sex-specific approaches are already a best-practice in gender-based violence prevention programming. See below for a few reviews.

Vladutiu CJ, Martin SL, Macy RJ. College-or university-based sexual assault prevention programs: a review of program outcomes, characteristics, and recommendations. Trauma, Violence, & Abuse. 2011;12(2):67–86.

Brecklin LR, Forde DR. A meta-analysis of rape education programs. Violence Vict. 2001;16(3):303–21.

Gibbons R, Evans J. The evaluation of campus-based gender violence prevention programming: what we know about program effectiveness and implications for practitioners. Retrieved from National Online Resource Centre on Violence Against Women’s website: [http://www](http://www/) vawnet org. 2013.

Although the majority of the reviewed literature is on workplace harassment (which is the majority of literature on sexual harassment so this is understandable), the authors very briefly mention that they are going to examine harassment in the context of online dating. They include a citation to three studies in that area. This is certainly a potential contribution as research has not thoroughly explored harassment in online contexts. However, there are numerous studies in the area of victimization facilitated by online dating, including perceptions of victimization, reporting, and sex differences. While not always restricted solely to harassment, many of these studies consider a range of behaviors that are considered sexual violence and harassment. The authors do not provide justification for their contribution above and beyond this literature. A few citations are below –

***Thank you for pointing this out. We agree that there is indeed a rich body of literature on online sexual violence and it was remiss not to mention these within our paper. We have corrected this and clarified that our goal is to take the next steps suggested by these and other papers to focus on that which may inform intervention development (p7).***

Choi, E. P. H., Wong, J. Y. H., & Fong, D. Y. T. (2018). An emerging risk factor of sexual abuse: the use of smartphone dating applications. Sexual Abuse, 30(4), 343–366.

Clevenger, S. L., Navarro, J. N., & Gilliam, M. (2018). Technology and the endless “cat and mouse” game: A review of the interpersonal cybervictimization literature. Sociology Compass, 12(12), 1–13.

Douglass, C. H., Wright, C. J., Davis, A. C., & Lim, M. S. (2018). Correlates of in-person and technology-facilitated sexual harassment from an online survey among young Australians. Sexual Health, 15(4), 361–365.

Henry, N., & Powell, A. (2018). Technology-facilitated sexual violence: A literature review of empirical research. Trauma, Violence, & Abuse, 19(2), 195–208.

March, E., Grieve, R., Marrington, J., & Jonason, P. K. (2017). Trolling on Tinder®(and other dating apps): Examining the role of the Dark Tetrad and impulsivity. Personality anD Individual Differences, 110, 139-143.

Phan, A., Seigfried-Spellar, K., & Choo, K. K. R. (2021). Threaten me softly: A review of potential dating app risks. Computers in Human Behavior Reports, 3, 100055.

Powell, A., & Henry, N. (2019). Technology-facilitated sexual violence victimization: Results from an online survey of Australian adults. Journal of Interpersonal Violence, 34(17), 3637–3665.

In sum, the authors do not provide adequate justification for this study. I do think they have the potential to make an important contribution if reconceptualized as the data collected seem to be novel. For example, the authors have a wide variety of vignettes that they include in their measure of harassment. Teasing those out to examine how definitions vary across different kinds of behavior and gender-differences in those perceptions would be a study that contributes to the harassment literature and the literature on online victimization, which often is narrowly focused on specific forms of victimization (e.g., cyberstalking).

***This is an interesting suggestion and we have now performed analyses and discussed findings of the vignettes individually to give the reader a better feel for the OD-SHAM and to make its components and summary statistics more transparent (pp 11-17).***

Likewise, the authors include personality scales that are not often considered (but see March et al above), perhaps more development around why those matter and nuanced analyses of those as they relate to harassment and outcomes would inform our current prevention programming.

***We have added to the introduction a discussion of traits associated with sexual harassment in other research and an explanation for the measures used within our study (p8). We also expanded on how revealing associated traits may aid intervention development in the final discussion (p23).***
