# Supplementary material for: The effects of sex and outcome expectancies on perceptions of sexual harassment
Source: PLoS One. 2021 Dec 15;16(12):e0261409. doi: 10.1371/journal.pone.0261409 (PMC8673621; doi:10.1371/journal.pone.0261409)
Supplement: Attachment — Submitted filename: Response to Reviewers.docx [file pone.0261409.s004.docx]

**17/11/2021**

**Response to reviewers**

***Thank you for giving us the opportunity to make these additional changes following our revision of our manuscript: The Effects of Sex and Outcome Expectancy on Perceptions of Sexual Harassment. We have tried to address all comments made and, as a result, we feel that the manuscript has greatly improved.***

COMMENTS TO THE AUTHOR

**Associate Editor:**

Thank you for submitting your manuscript to PLOS ONE. After careful consideration, we feel that it has merit but does not fully meet PLOS ONE’s publication criteria as it currently stands. Therefore, we invite you to submit a revised version of the manuscript that addresses the points raised during the review process.

Specifically, the same peer reviewer that provided feedback on the last version submitted a new review of the revised manuscript, and I again independently reviewed the revision. As noted by the reviewer, the revisions were highly responsive to comments and provided a balanced overview of the theoretical landscape on harassment research, including highlighting the sex-specific nature of many harassment trainings currently. The revision also provided more information about the development of the vignette paradigm, and other comments were thoroughly addressed. The reviewer had a few more comments during this round of review that I will not repeat here but are worthy to address in another revision before the paper is deemed ready for publication.

One other note, relevant to the reviewer’s suggestion to follow APA or other reference styles guidelines regarding use of language in gender studies, it seems that “gender” rather than “sex” differences are the focus of your study, and thus, this may be another language change to consider: <https://apastyle.apa.org/style-grammar-guidelines/bias-free-language/gender>.

***This is an important point and we have made the effort to ensure our wording is appropriate throughout. We asked participants for both their assigned biological sex and gender identity, but the analyses groups were formed based on biological sex as opposed to gender. We believe this is fitting with the evolutionary theory applied within the discussion. However, we have clarified this within the text by clearly stating we used “assigned biological sex” for analyses (p.10). We also reran the analyses using gender identity which was not qualitatively different and would not change the overall interpretation of the results. Changes to findings based on gender have now been noted where relevant throughout the results section.***

**Reviewer #1:**

**Literature Review**
- I found the review of the literature in the revision to be much more balanced. The authors did a commendable job integrating a wider range of theoretical perspectives that research has taken for sexual harassment. The linking aspect of these perspectives of sexual harassment as goal-oriented behavior sets up their study well.
The rationale for the hypotheses are very brief. The idea that sexual harassment would be associated with outcome expectancy is discussed. The second two ideas – moderation effects and sex differences – need some further justification and elaboration.

***Thank you for pointing this out, we now realise there was an imbalance between our hypotheses’ rationale. We have expanded on why moderation effects and sex differences are part of our hypotheses and believe this section will be much clearer to the reader as a result (pp.8-9).***

Likewise, while the authors discuss OEs at length, they do not discuss the importance of defining behaviors as harassment and why linking those two concepts together is important. While it may seem obvious as to why it matters, it does not come across in the manuscript.

***This is a good point. As stated by the reviewer, it may seem obvious to us as the writers, but we should not assume the same of all readers. We now have a paragraph discussing this matter in the introduction and believe this makes the overall point of our research much more apparent (p.4).***

The discussion of defining behaviors as harassment should also include a sex-specific component given H1 is hypothesizing sex differences in definition that are not discussed in the literature review.

***As suggested, we have noted sex differences in perceptions of sexual harassment behaviours in this new paragraph.***

**Results**
- The comparisons in Table 1 comprise many comparison statistical tests between men and women. It does not appear that the significance of the p-value was adjusted to account for this.

***We have corrected this oversight throughout all analyses which makes the subsequent findings that much more robust.***

**Minor and Miscellaneous**
In line with recent guidance from several reference styles (e.g., APA), consider using “men” and “women” as nouns and “males” and “females” as adjectives to avoid biased language. <https://apastyle.apa.org/style-grammar-guidelines/bias-free-language/gender>

***Thank you for providing these guidelines. We have made the necessary corrections within the manuscript.***

Please review your reference list to ensure that it is complete and correct. If you have cited papers that have been retracted, please include the rationale for doing so in the manuscript text, or remove these references and replace them with relevant current references. Any changes to the reference list should be mentioned in the rebuttal letter that accompanies your revised manuscript. If you need to cite a retracted article, indicate the article’s retracted status in the References list and also include a citation and full reference for the retraction notice.

***We have checked the references and, whilst none of the papers appear to have retracted, we did find some links that have stopped working since we wrote them and one paper that was a preprint but has now been published. We have updated these accordingly and hope that this addresses the matter.***
